# Supplementary material for: Modeling Membrane Morphological Change during Autophagosome Formation
Source: iScience. 2020 Aug 15;23(9):101466. doi: 10.1016/j.isci.2020.101466 (PMC7479497; doi:10.1016/j.isci.2020.101466)
Supplement: Document S1. Transparent Methods and Figures S1–S7 [file mmc1.pdf]

iScience, Volume *23*

Supplemental Information

Modeling Membrane Morphological Change during Autophagosome Formation

Yuji Sakai, Ikuko Koyama-Honda, Masashi Tachikawa, Roland L. Knorr, and Noboru Mizushima

Transparent Methods

Geometrical parameters of the model membrane

The geometry of the outer and inner membranes is modeled as a part of an ellipsoid structure with a bending angle α, while the rim geometry is modeled as a part of a torus with a fixed minor radius r (Figure 2). In the ellipsoid approximation, the total curvature J_+ (J_-) and the surface element A_+ (A_-) of the outer (inner) membrane is given by

$$J_\pm = \frac{\gamma}{R_\pm}\frac{cos^2 t + \gamma^2 sin^2 t + 1}{(cos^2 t + \gamma^2 sin^2 t)^{3/2}}, \tag{S1}$$

$$dA_\pm = 2\pi R_\pm^2 \sin t \sqrt{cos^2 t + \gamma^2 sin^2 t}\, dt, \tag{S2}$$

where $R_\pm = R \pm r$ is the radius of the short axis of the outer (inner) membrane. The integration variable t runs along the interval $[0, \alpha]$, and γ is the aspect ratio of the long axis to the short axis (Figure 2). Note that γ is undefined for α = 0. In the torus approximation, the total curvature and the surface element of the rim membrane are respectively given by

$$J_r = \frac{1}{r} - \frac{cos\,\varphi}{\Delta - r\,cos\,\varphi}, \tag{S3}$$

$$dA_r = 2\pi r(\Delta - r\,cos\,\varphi)d\varphi, \tag{S4}$$

where $\Delta = R\sin\alpha$ is the radius of the aperture (Figure 2). The integration variable φ runs along the interval $[\alpha - \pi/2, \alpha + \pi/2]$.

The rim area is $A_r = 2\pi r(\Delta\pi + 2r\,cos\,\alpha)$, and the outer (+) and the inner (−) membrane areas are

$$A_\pm = 2\pi R_\pm^2 F(\gamma, \alpha), \tag{S5}$$

$$F(\gamma, \alpha) = \int_{cos\,\alpha}^{1} \sqrt{\gamma^2 + (1-\gamma^2)x^2}dx\,, \tag{S6}$$

where $F(\gamma, \alpha)$ is the form factor of the ellipsoid.

Equilibrium conditions

We consider the membrane morphology and the distribution of curvature generators, which are obtained by minimizing the free energy, Equation (1), for a given total membrane area, A, and the abundance of the curvature generators, A_ϕ. The optimal membrane radius R^*, the bending angle α^*, the aspect ratio γ^*, and the area fraction of the curvature generators at each region $(\phi_r^*, \phi_+^*, \phi_-^*)$ are obtained by minimization,

$$F_{tot}(\{X_i^*\}) = min\{F_{tot}(\{X_i\})\}, \tag{S7}$$

with $X_i = (\alpha, \gamma, R, \phi_r, \phi_+, \phi_-)$. Theses parameters satisfy the constraints

$$A_r + A_+ + A_- = A, \qquad A_r\phi_r + A_+\phi_+ + A_-\phi_- = A_\phi. \tag{S8}$$

If curvature generators on the membrane can be exchanged with those in the surrounding environment (e.g., cytosol), the equilibrium state is realized by minimizing the grand potential, Equation (5), for a given total membrane area A,

$$\Omega(\{X_i^*\}) = min\{\Omega(\{X_i\})\}, \tag{S9}$$

with $X_i = (\alpha, \gamma, R, \phi_r, \phi_+, \phi_-)$. The abundance of curvature generators, A_ϕ, is variable in this case.

Effects of two different types of curvature generators

In the presence of a second type of curvature generator with a different spontaneous curvature ζ_j, the spontaneous curvature of the bending energy and the partitioning entropic energy in the model are slightly modified. The spontaneous curvature, Equation (3), is modified into

$$\bar{J} = \sum_j \frac{1}{2}\zeta_j \phi_{i,j}\,, \tag{S10}$$

where $\phi_{i,j}$ is the area fraction of type-j curvature generators in area i. The partitioning entropic energy, Equation (4), is modified into

$$F_{part} = -k_B T \sum_{i=\pm,r}\left(\sum_j \phi_{i,j} ln\phi_{i,j} + \left(1 - \sum_j \phi_{i,j}\right) ln\left(1 - \sum_j \phi_{i,j}\right)\right)\frac{A_i}{a_\phi}. \tag{S11}$$

Two types of curvature generators with different spontaneous curvatures ζ_1 and ζ_2 are considered for simplicity. Extension to more types of curvature generators is straightforward.

Evaluation of the membrane area and the bending angle from *in vivo* experiments

The membrane area and the bending angle were calculated from the image of mRubby3-LC3B and GFP-ATG2A. The contour of membranes was extracted from the region labeled with mRubby3-LC3B. We added a width of 0.2 μm to the contour, which came from the diffraction limit (red in Figure S7). We fitted the data point with an ellipse

$$\left(\frac{(x-x_0)\cos\theta + (y-y_0)\sin\theta}{R}\right)^2 + \left(\frac{(x-x_0)\sin\theta + (y-y_0)\cos\theta}{\gamma R}\right)^2 = 1 \tag{S12}$$

and obtained the radius R, the aspect ratio γ, the origin (x_0, y_0), and the orientation θ. The bending angle was obtained from the origin and overlapping region of the mRubby3-LC3B contour and GFP-ATG2A contour, to which a width of 0.2 μm was added (green in Figure S7).

For the disk shape ($\alpha = 0$), the membrane area is given by

$$A = 2\pi R^2 + 2\pi^2 rR + 4\pi r^2, \tag{S13}$$

with the rim radius $r = 10\,nm$. By fitting the shape labeled with mRubby3-LC3B with a sphere, the radius R was obtained, and then the area A was obtained. For the cup shape ($\alpha > 0$), the membrane area is given by

$$A = 4\pi(R^2 + r^2)F(\gamma, \alpha) + 2\pi r(\pi R\sin\alpha + 2r\cos\alpha), \tag{S14}$$

$$F(\gamma,\alpha) = \int_{cos\,\alpha}^{1} \sqrt{\gamma^2 + (1-\gamma^2)x^2}dx\,. \tag{S15}$$

By fitting the shape of the area labeled with mRubby3-LC3B with a part of an ellipsoid, the bending angle α, the radius R, and the aspect ratio γ were obtained, and then, the area A was obtained.

Plasmids

Full-length cDNA of rat microtubule-associated protein 1 light chain 3B (LC3B, GenBank: NP_074058) was subcloned into the pMRX-IP vector (Saitoh et al., 2003), which was generated from pMXs (Kitamura et al., 2003), together with DNA encoding codon-optimized mRuby3 (modified from pKanCMV-mClover3-mRuby3; 74252: Addgene) (Matsui et al., 2018). The pMRX-IP-GFP-ATG2A vector was previously described (Velikkakath et al., 2012).

Cell culture

Mouse embryonic fibroblasts (MEFs) were cultured in Dulbecco's modified Eagle's medium (DMEM: D6546, Sigma-Aldrich) supplemented with 10% fetal bovine serum (172012, Sigma-Aldrich), and 2 mM L-glutamine (25030-081, Gibco) in a 5% CO_2 incubator. For the starvation treatment, cells were washed twice and incubated in amino acid-free DMEM (048-33575, Wako Pure Chemical Industries) without serum.

Retroviral preparation and establishment of stable cell lines

Stable cell lines were generated by a retrovirus-mediated transformation method as previously described (Nishimura et al., 2013).

Fluorescence microscopy

Cells stably expressing mRuby3-LC3B and GFP-ATG2A were subjected to live-cell fluorescence imaging using the DeltaVison Elite microscope (GE Healthcare Life Science) equipped with a PLAPON 60XO oil-immersion objective lens (NA 1.42, Olympus) and a cooled-CCD camera (CoolSNAP HQ2, Photometrics). During live-cell imaging, the dish was mounted in a chamber (INUB-ONI-F2, TOKAI HIT) to maintain the incubation conditions at 37°C and 5% CO_2. Images were acquired at intervals of 10 s for 30 min.

Supplemental Figures

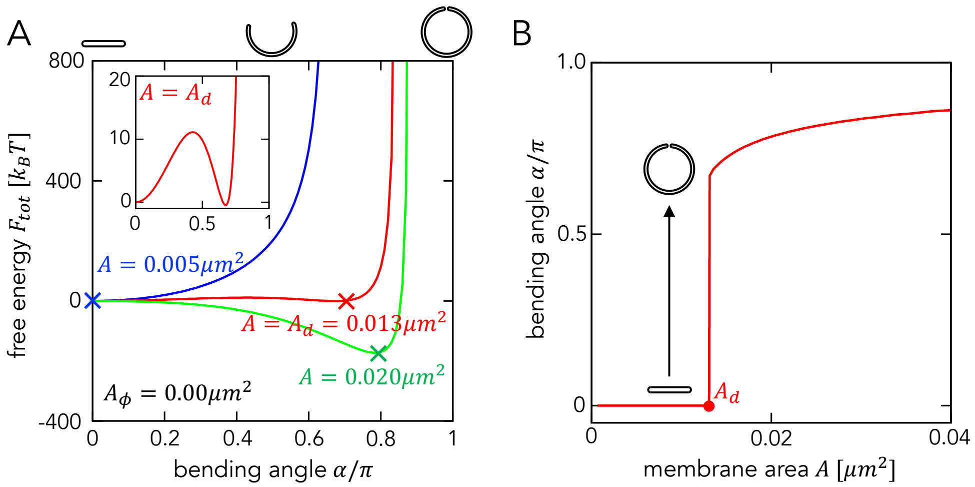

Figure S1. Relationship between the bending angle and the total membrane area with no curvature generators, related to Figure 4. (A) The total free energy as a function of the bending angle α for the membrane area $A = 0.005, 0.013$ (A_d), and 0.020 μm^2, where the aspect ratio γ is taken to minimize the free energy at each α. The minima are marked by × symbols. The inset shows the enlarged view of the free energy at $A = A_d$. (B) The bending angle α as a function of the membrane area A. The transition points (A_d) are marked by ● symbols.

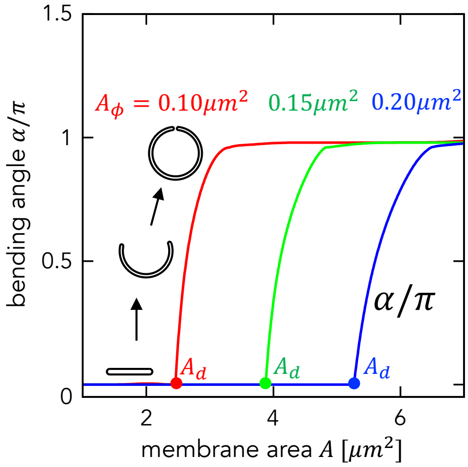

Figure S2. The bending angle in spherical geometry with a fixed aspect ratio $\gamma = 1$, related to Figure 4. The bending angle in spherical geometry with a fixed aspect ratio $\gamma = 1$. The bending angle as a function of the membrane area A for $A_\phi = 0.1$ (red), 0.15 (green), and $0.2\ \mu m^2$ (blue). The transition points (A_d) are marked by ● symbols.

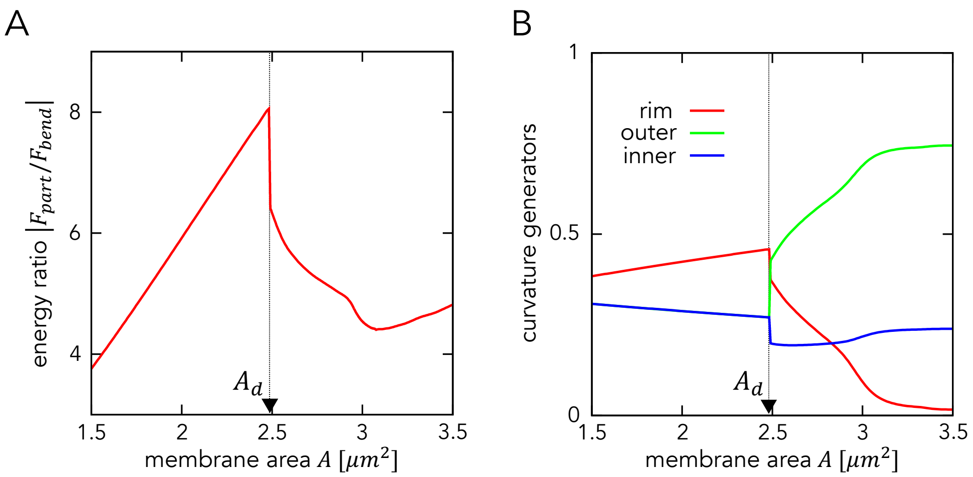

Figure S3. Relationship between the energy ratio and curvature generator membrane area, related to Figure 5. (A) Ratio of the partitioning entropic energy F_{part} over the bending energy F_{bend} as a function of the membrane area A for $A_\phi = 0.1\ \mu m^2$. (B) Ratio of curvature generator abundance in each region. The total abundance is fixed at $A_\phi = 0.1\ \mu m^2$.

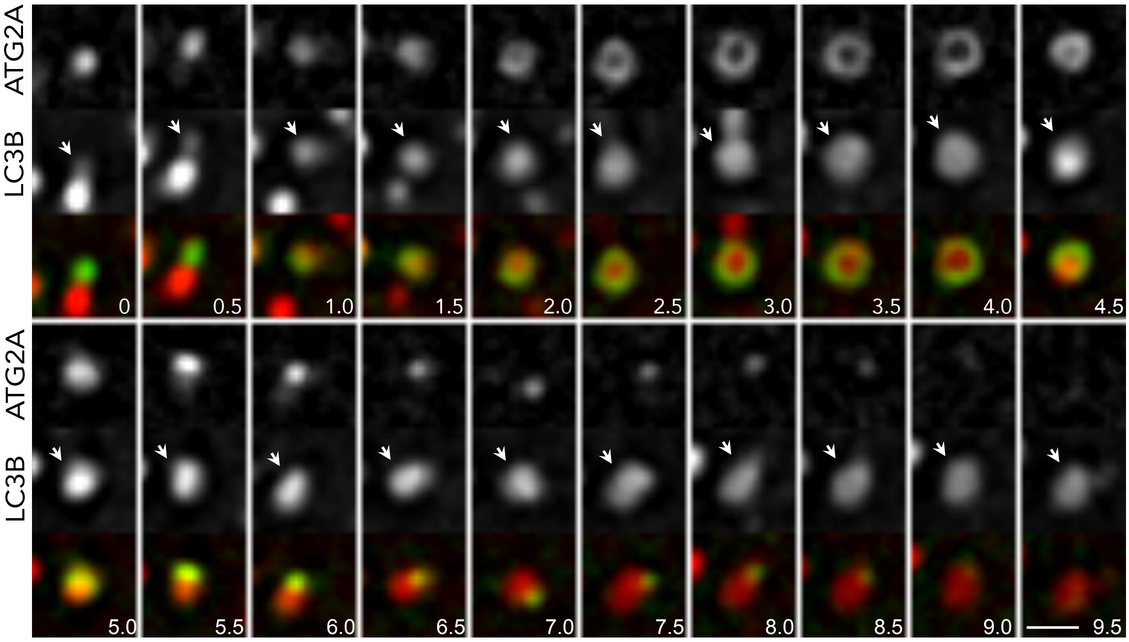

Figure S4. Time-lapse imaging of autophagosome formation, related to Figure 7. Mouse embryonic fibroblasts (MEFs) expressing mRuby3-LC3B (red) and GFP-ATG2A (green) were starved, and images were captured every 30 s; scale bar, 1 μm.

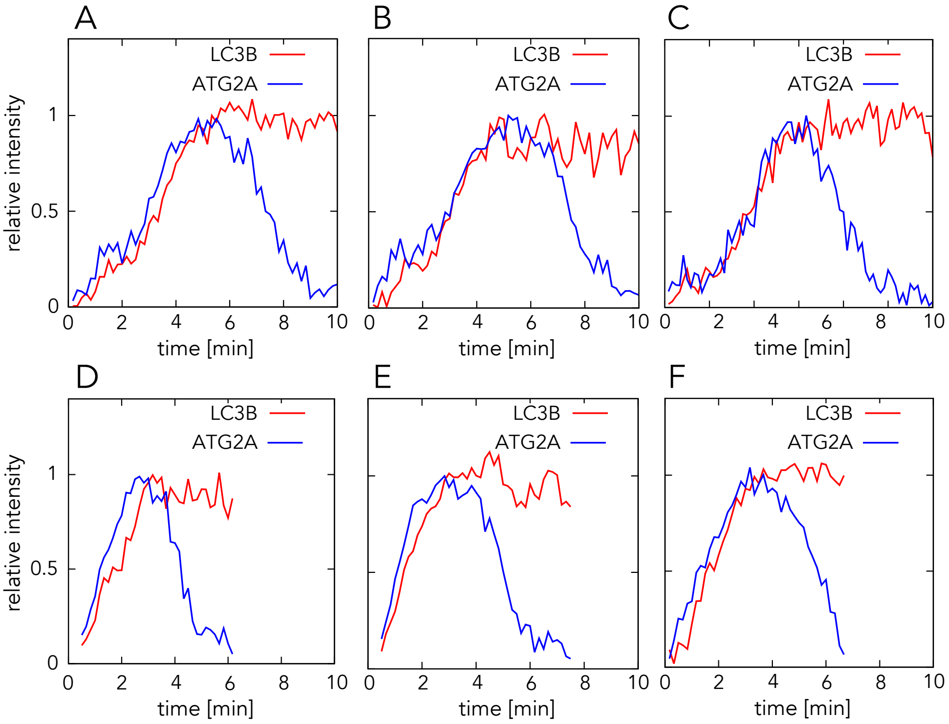

Figure S5. Quantification of LC3B and ATG2A intensities during autophagosome formation, related to Figure 7. The time course of the total fluorescent intensity of mRuby3-LC3B (red) and GFP-ATG2A (blue) of each structure is shown throughout autophagosome formation in starved mouse embryonic fibroblasts (MEFs; shown as percentage of maximum intensity). Six independent cases are shown in A–F. Panel A shows the results in Figure S2.

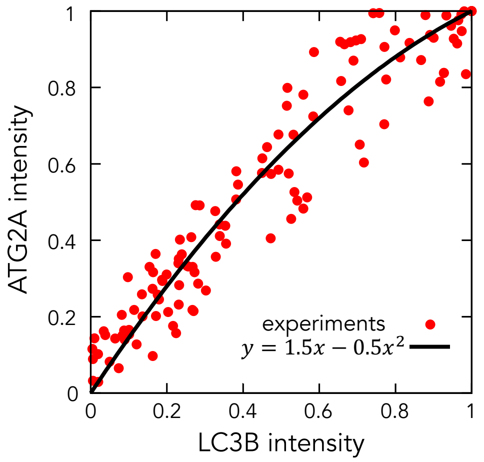

Figure S6. Correlation between total mRuby3-LC3B and GFP-ATG2A intensities of each structure during the initial phase of autophagosome formation (until their intensities reach a plateau or peak, related to Figure 7. Each data point is taken from the results shown in Figure S3. The solid line indicates the second-order polynomial fit of the data.

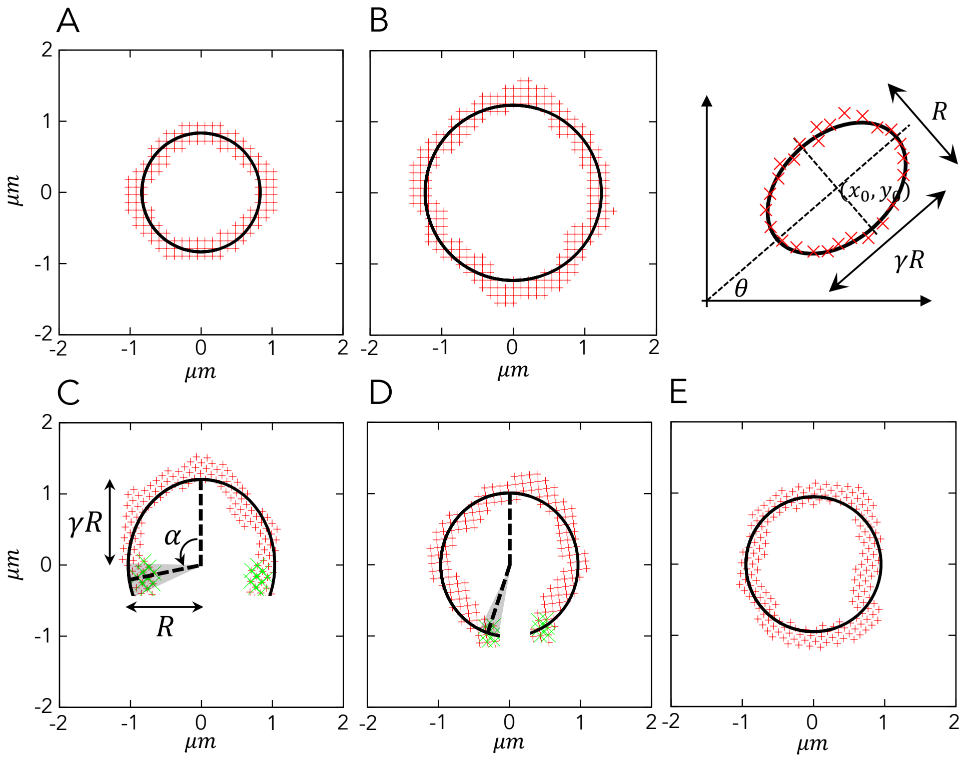

Figure S7. An example of morphological changes during autophagosome formation, related to Figure 7. The images in Figure 7A were fitted with a part of an ellipsoid, where the axis dimension is measured in μm. Here the membrane is assumed to take a disk shape in A and B and a cup shape in C–E.

Supplemental References

Kitamura, T., Koshino, Y., Shibata, F., Oki, T., Nakajima, H., Nosaka, T., and Kumagai, H. (2003). Retrovirus-mediated gene transfer and expression cloning: Powerful tools in functional genomics. Exp. Hematol. 31(11), 1007-14.

Matsui, T., Jiang, P., Nakano, S., Sakamaki, Y., Yamamoto, H., and Mizushima, N. (2018). Autophagosomal YKT6 is required for fusion with lysosomes independently of syntaxin 17. J. Cell Biol. 217(8), 2633–2645.

Nishimura, T., Kaizuka, T., Cadwell, K., Sahani, M.H., Saitoh, T., Akira, S., Virgin, H.W., and Mizushima, N. (2013). FIP200 regulates targeting of Atg16L1 to the isolation membrane. EMBO Rep. 14(3), 284-91.

Saitoh, T., Nakayama, M., Nakano, H., Yagita, H., Yamamoto, N., and Yamaoka, S. (2003). TWEAK induces NF-κB2 p100 processing and long lasting NF-κB activation. J. Biol. Chem. 278(38), 36005-12.

Velikkakath, A.K.G., Nishimura, T., Oita, E., Ishihara, N., and Mizushima, N. (2012). Mammalian Atg2 proteins are essential for autophagosome formation and important for regulation of size and distribution of lipid droplets. Mol. Biol. Cell. 23(5), 896-909.
